# Investigating the Relationships Between Three Important Functional Tasks Early After Stroke: Movement Characteristics of Sit-To-Stand, Sit-To-Walk, and Walking

**DOI:** 10.3389/fneur.2021.660383

**Published:** 2021-05-14

**Authors:** Elizabeth Ann Chandler, Thomas Stone, Valerie Moyra Pomeroy, Allan Brian Clark, Andrew Kerr, Phillip Rowe, Ukadike Chris Ugbolue, Jessica Smith, Nicola Joanne Hancock

**Affiliations:** ^1^Faculty of Medicine and Health Sciences, University of East Anglia, Norwich, United Kingdom; ^2^Department of Clinical Engineering (Addenbrookes), Cambridge University Hospitals National Health Service Foundation Trust, Cambridge, United Kingdom; ^3^National Institute for Health Research Brain Injury MedTech Cooperative, Cambridge, United Kingdom; ^4^Department of Biomedical Engineering, Faculty of Engineering, University of Strathclyde, Glasgow, United Kingdom; ^5^School of Science and Sport, University of West of Scotland, Hamilton, United Kingdom; ^6^Department of Performance Analysis, English Institute of Sport, Sheffield, United Kingdom

**Keywords:** stroke, walking, sit-to-stand, sit-to-walk, measurement, biomechancis, movement fluidity, neuromuscular recovery

## Abstract

**Background:** Walking, sit-to-stand (STS) and sit-to-walk (STW) are all considered important functional tasks in achieving independence after stroke. Despite knowledge that sensitive measurement of movement patterns is crucial to understanding neuromuscular restitution, there is surprisingly little information available about the detailed biomechanical characteristics of, and relationships between, walking, sit-to-stand and sit-to-walk, particularly in the important time window early after stroke. Hence, here, the study aimed to:

1. Identify the biomechanical characteristics of and determine any differences in both movement fluidity (hesitation, coordination and smoothness) and duration of movement phases, between sit-to-stand (STS) and sit-to-walk (STW) in people early after stroke.
2. Determine whether measures of movement fluidity (hesitation, coordination, and smoothness) and movement phases during sit-to-stand (STS) and/or sit-to-walk (STW) are correlated strongly to commonly used measures of walking speed and/or step length ratio in people early after stroke.

**Methods:** This study consisted of secondary data analysis from the SWIFT Cast Trial. Specifically, we investigated movement fluidity using established assessments of smoothness, hesitation and coordination and the time duration for specific movement phases in a group of 48 people after stroke. Comparisons were made between STS and STW and relationships to walking measures were explored.

**Results:** Participants spent significantly more time in the initial movement phase, flexion momentum, during STS [mean time (SD) 1.74 ±1.45 s] than they did during STW [mean time (SD) 1.13 ± 1.03 s]. STS was also completed more smoothly but with more hesitation and greater coordination than the task of STW. No strong relationships were found between movement fluidity or duration with walking speed or step length symmetry.

**Conclusions:** Assessment of movement after stroke requires a range of functional tasks and no one task should predominate over another. Seemingly similar or overlapping tasks such as STS and STW create distinct biomechanical characteristics which can be identified using sensitive, objective measures of fluidity and movement phases but there are no strong relationships between the functional tasks of STS and STW with walking speed or with step-length symmetry.

## Introduction

Regaining the ability to walk again after stroke is a priority for stroke survivors (1). Current evidence indicates that task-specific activity i.e., practice of functional walking activity, is the best approach to promoting recovery, where recovery is defined as “the extent to which body structure and functions, as well as activities, have returned to their pre-stroke state” (2). But provision of evidenced-based task-specific walking practice is challenging, especially for people with substantial motor impairments. This challenge is particularly pertinent early after stroke when it is important to provide intensive input, focused on restitution of neuromuscular function, whilst people are still in the period of injury-induced neuroplasticity (3, 4). Other rehabilitation tasks are often used when walking rehabilitation is not possible in everyday therapy.

For example, clinical therapy early after stroke often centers on perhaps less challenging, but nonetheless important, functional activities such as sit-to-stand (STS) and sit-to-walk (STW). STS is a relatively simple, symmetrical movement, easy to train as a single task, and is important for independence in activities of daily living such as washing and dressing (5, 6). Conversely, the associated functional task of sit-to-walk (STW) is a more complex, asymmetric activity that combines rising from sitting and gait initiation, *via* fluent movement transitions, to enable speed and efficiency of movement. Indeed, fluidity of STW could be seen as an expression of intact motor control and, like walking, this complex movement is challenging for people with motor deficit after stroke (7). As such, it is possible that STW may be associated with other important dynamic functions that require fluid movement between transitions, such as walking, and, in particular, walking that requires adaptation of parameters to meet environmental demands (8). Certainly, work on a previously developed Fluidity Index (9) suggested an association with fluidity measures during rising to walk and gait speed, though this was not tested statistically, and this same work found a significant correlation between overall movement duration and gait speed. It should be noted that the Index used in this work (9) was based on Center of Mass (CoM) velocity in one direction only. STS duration has also been shown to relate to spatiotemporal parameters of walking including walking speed but not to symmetry (10) or more complex measures of fluidity (6). In order to more fully understand the potential relationships between these important, commonly adopted functional tasks more fully, a detailed assessment using measures that reflect the complexity of the tasks, is required.

However, despite the established importance of these key functional tasks- STS, STW, and walking- and some indication of relationships between them, detailed assessment of their biomechanical characteristics in the same group of people in the important time window early after stroke remains sparse, both in research and clinical practice. An understanding of such characteristics is crucial to understanding neuromuscular restitution (11). Sensitive, objective measurement of movement patterns is key to this understanding and can be achieved using kinetics and kinematics during functional activity (11, 12), yet, other measures predominate; walking speed is a current foremost measure of functional ability (13). This may not be the appropriate measure to investigate neuromuscular restitution, as observation indicates that people using compensatory movement patterns- “neuromuscular substitution”- can walk at the same speed as people who do not (13). Other temporal-spatial characteristics of gait are also measured in some trials. But they too may not be measuring neuromuscular restitution alone, although derived measures of symmetry such as step length ratio could be indicative of change in movement patterns.

At present, there is little, if any information available on the best measures to assess neuromuscular restitution required for performance of important functional tasks (14). Nor has sufficient consideration been given to how neuromuscular improvement in one functional task may, or may not, generalize beyond that task e.g., from STS to STW, and STW and/or STS to walking. This is potentially important for future clinical recommendations—if walking speed and/or step length ratio are strongly correlated to one or more components of movement fluidity in other commonly trained functional activity such as STS and STW, then measurement of the latter could be superfluous, Furthermore, training of STS and STW in the early stages after stroke when walking practice is challenging, could improve walking parameters. And then, if there is a strong correlation between movement fluidity components during STS and STW after stroke then it is not essential to use both mobility tasks.

Therefore, to identify relevant biomechanical characteristics of neuromuscular restitution, according to rehabilitation science consensus (11) we should firstly establish and compare movement fluidity measures (hesitation, coordination, and smoothness) and/or measures of timing within movement phases from a set of functional tasks after stroke, such as STS and STW, not just walking. Then, the relationship between those measures and more commonly used clinical measures of walking should be explored. Such detailed investigation of these issues are warranted before further steps toward future clinical recommendations on the type of training to be used can be made (11).

Hence, the overarching hypothesis driving the study reported here is that measurement of fluidity derived from kinematic and kinetic variables during the functional tasks of STS, STW and walking show strong association. In order to investigate this hypothesis, the specific aims of the study reported here were:

1. To firstly identify the detailed biomechanical characteristics of, and determine any differences in, both movement fluidity (hesitation, coordination and smoothness) and duration of movement phases between sit-to-stand (STS) and sit-to-walk (STW) in people early after stroke.
2. To then determine whether measures of movement fluidity (hesitation, coordination, and smoothness) and movement phases during sit-to-stand (STS) and/or sit-to-walk (STW) are correlated strongly to commonly used measures of walking speed and/or step length ratio in people early after stroke.

## Materials and Methods

### Design

This was an observational study comparing the same group of participants early after stroke during sit-to-stand (STS) sit-to-walk (STW) and walking. The study aims here were addressed by secondary data analysis of movement data collected during the SWIFT Cast Trial (15).

### Participants

People were included as participants in the primary SWIFT Cast Trial (15) if they were:

over 18 years old;
between 3 and 42 days after stroke, either infarct or hemorrhage;
considered to be fit for rehabilitation, having peripheral oxygen saturations 90%+ on air, resting pulse <101 beats/min;
able to take at least three steps with abnormal initial foot contact and/or decreased ability to take full body weight through the paretic lower limb during stance; with the assistance of up to two people if required;
able to follow a 1-stage command; and
free from contractures or loss of skin integrity in lower limb.
For inclusion in the secondary analysis presented here, participants were those who met the above criteria, 1–7, and who were:
able to complete a STS and STW task at the outcome measurement time point (~6 weeks after start of the intervention phase) without physical assistance from another person, object or aid (e.g., walking stick).

### Data Collection

Kinematic and kinetic data were collected in the movement laboratories of the University of Strathclyde and the University of East Anglia. Vicon motion capture cameras (Oxford Metrics, Oxford, UK) were used to capture 3D trajectories of 48, 14 mm reflective markers attached to the body at anatomical locations in accordance with a bespoke biomechanical model that used a combination of cluster and anatomical markers (16). This biomechanical model has also been validated for use among stroke patients (17). Marker trajectory data were sampled at 100 Hz. Embedded force plates were used to record ground reaction forces sampled at 1,000 Hz at the University of Strathclyde (Kistler Instrumente AG, Switzerland) and 2,000 Hz at the University of East Anglia (Bertec, Columbus, OH).

Participants wore tight-fitting Lycra shorts and vest along with comfortable flat shoes. The STS and STW movements were completed from a height adjustable plinth, setup to allow the participant to sit with their feet flat on the floor, hips and knees as close to 90 degrees as possible. Each foot was positioned on an embedded force plate, approximately shoulder width apart and facing the direction of progression. Participants were asked not to use their upper limbs to assist them in the task. However, they were not prevented from using their upper limbs to steady themselves when they felt unsafe as they rose. For the analysis presented here, these trials were included as they represent the pragmatic movement strategy adopted by these participants who were representative of the clinical population. In effect, a quarter of the participants steadied themselves during rising in one or more trials. For each task, a minimum of three and a maximum of six repetitions of each task (trials) were undertaken.

#### STS Task

Participants were instructed to stand up as soon as they heard a buzzer, and remain standing until they saw a red light accompanied by a second buzzer, at which point they sat down.

Sufficient time was given between buzzers to enable a stable upright standing position to be achieved, determined by researcher observation.

#### STW Task

Participants were instructed to go and pick up a cup from a table as soon as they heard the buzzer. This instruction was designed to elicit a voluntary STW movement. The distance between the and the participants' seated position was standardized at 3 m.

Data collection and analysis for walking speed and walking step length symmetry is described in earlier publications (18, 19). In brief, participants walked at a self-selected speed along a 6 m mat which was marked with lines 1, 5, and 10 cm apart. Circular black and white markers were placed over each participant's skin to mark the joint centers of the hip, knee, and ankle. High speed video cameras (EXFH20, Casio, Tokyo, Japan) were used to record the participant walking and additionally to detect the timing of when the participant crossed into and out of the 6 m space. The start and end times were identified by a flash emitted from a light source when infra-red beams at the start and end of the mat were broken by the participant passing through. Video data was processed using Pro-trainer 10.1 (Sports Motion Inc., CA, USA) to determine step times and to extract step lengths using the markings on the mat. Step length symmetry values were calculated using the equation.

$$\begin{array}{l} Step\;Length\;Symmetry\;=\;\frac{2P}{P+LP}-\;1 \end{array}$$

where P = Paretic leg and LP = Less paretic leg values. A positive value implies longer step length on the paretic leg, and a negative value longer length in the non-paretic.

### Data Processing

Kinematic and kinetic data were synchronized using Vicon Nexus software (Oxford Metrics, Oxford, UK). Marker trajectories were filtered using a Woltring filter with a predicted mean square error of 20 mm. Model outputs were filtered using a low pass (cut off frequency 6 Hz) sixth order Butterworth filter.

STW gait events of “foot strike” and “foot off” were independently marked and verified by two researchers. Where available, force-plate data were used to further verify the time-position of events. Marker trajectories and model outputs were exported and custom scripts in Python (Python Software Foundation, www.python.org) were used for all further analyses.

### Movement Phases

Movement phases were assessed by the total time taken for STS and STW tasks, along with timing of specific within-task movement phases as described by Kerr et al. (20). These movement phases were adapted here, as data collection did not include kinematic data to mark seat off, and due to difficulties identifying gait initiation in this group of people early after stroke (see phase descriptions below). Direct comparison between STS and STW can only be made for Phases 1 and 2 which are shared by both STS and STW. Phase 3 begins with the same biomechanical event for STS and STW, but due to the different nature of the tasks, the end event differs. The authors considered that to exclude Phase 3 would be an omission so comparison is included; however, it is most useful for consideration in addressing aim two.

Phase 1, *flexion momentum*, began with initiation of movement of the clavicle marker and continued until peak vertical force was reached. Phase 2, *seat-off* , was defined as the time between peak vertical force and peak vertical velocity of the clavicle marker. Phase 3, *extension momentum*, began at peak vertical velocity of the clavicle marker and ended at (i) maximum height of the clavicle marker for STS or (ii) foot off during the first swing phase of gait for STW (unloading). Finally, Phase 4, *stance*, occurs in STW only. It denotes the time between foot off of initial swing phase, until the foot off of the opposite leg (the initial stance leg). As reported previously in this study population (21, 22), it was not possible to reliably identify the mediolateral ground reaction force denoting the start of gait initiation; foot off was therefore used to mark transition between Phases 3 and 4 during STW.

### Fluidity Measures

All fluidity measures for STS and STW- smoothness, hesitation and coordination, were calculated from time normalized data. For the purpose of this analysis, both tasks began with the initiation of movement. Initiation was defined here as the instance when the vertical velocity of the clavicle marker changed by more than 0.5 mms^−1^ from baseline and was sustained for at least 50 ms prior to the clavicle marker's minima position in the vertical plane. The movement cycles ended at the maximal peak of vertical displacement of the clavicle marker for STS and foot contact at the end of the second step i.e., foot contact of the original stance leg, for STW.

Previous studies have used model derived Center-of-Mass (COM) to calculate smoothness and hesitation; however, this requires full visibility of all tracking markers. Tasks which incorporate a sitting or flexed position present challenges for marker visibility; this, combined with the need for close supervision to maintain safety, resulted in some trials with missing marker position data. Gap filling interpolation methods are not applicable if the gap is at the beginning or end of the movement, or if gaps in the trajectory data are large. Hence, here we used the clavicle marker to track the fluidity of the trunk as it was reliably in view throughout trials. This simplified metric, when compared to COM, cannot fully account for the contribution of the upper limbs and head; nevertheless, it provides a useful and clinically applicable comparative measure as the trunk cannot act in isolation of the head and limbs. The sternum has previously been used to represent the COM during biofeedback to stroke survivors (23). Further, to check our decision, sternum, and clavicle positional data were compared to COM positions in 11 of the included participants for whom COM data was available. The magnitude of both COM, Sternum and Clavicle positional data was normalized and compared using the coefficient of determination which revealed an average correlation of the two signals of 95%.

#### Smoothness

Smoothness of the STS and STW tasks were defined according to the principles of Kerr et al. (24); where smoothness is derived from the rate of change of acceleration (jerk), calculated as the third time derivative of the horizontal position of the clavicle marker. The jerk signal was tested against a logic statement to count all instances when the signal was either (i) greater than the previous two samples and greater than the successive two samples, or (ii) less than the previous two samples and less than the subsequent two samples (24). Instances where the logic statement was met were defined as inflections in the jerk signal. Smoothness of the task was determined by the total inflection count, with a lower value indicating a smoother overall movement.

#### Hesitation

Hesitation of both STS and STW was measured as the percentage of normalized time between the maximum forward velocity and the maximum upward acceleration of the clavicle marker, where a low value indicates a fluid movement without hesitation. In contrast to previous publications (24, 25), here hesitation does not measure the depression in horizontal momentum. It was considered important to change the calculation for hesitation to provide an equitable measure between the tasks of STS and STW: STW is fundamentally about forward momentum, whereas STS is not.

#### Coordination

Coordination was also defined according to Kerr et al. (24). Two separate coordination values were calculated. Coordination One (C1) was derived from the temporal overlap between the knee and hip, in the sagittal plane, at the end of initial hip flexion and the start of knee extension; and Coordination Two (C2) derived from the temporal overlap between the knee and hip, in the sagittal plane at the end of hip extension and start of knee flexion on the initial step of STW (24). The events marking the start and end of hip and knee flexion were identified by first fitting a polynomial curve to the model derived data before calculating the differential values. The peaks in the resulting data describe the start and end events of hip and knee flexion. Previous studies have considered C1 of the paretic leg during STS (6) and C1 and C2 of the stepping leg during STW (24). For this analysis, C1 was calculated for both paretic and non-paretic legs during STS and STW tasks where marker visibility allowed. A lower value here indicates a more coordinated movement.

### Data and Statistical Analysis

The SWIFT Cast Trial did not find statistically significant differences between the experimental and control groups therefore, for addressing study aims here, participants were analyzed as a single group. Descriptive statistics were used to describe clinical characteristics of participants. Statistical analyses were performed using Stata 16.0/SE. A sample size calculation was not preformed due to this being a secondary analysis of an existing data set; a formal sample size calculation was carried out for the primary study (15).

Fluidity measures of smoothness, hesitation, and coordination were calculated per participant for all available trials along with total time to complete each task and duration of time spent in each defined movement phase. Repetitions of the STS and STW, respectively, were combined and the mean value calculated for each participant and task.

Paired *t*-tests were used to determine the differences between STS and STW (aim one) for:

a) fluidity measures; and
b) movement phase durations.

To determine whether measures of movement fluidity (hesitation, coordination and smoothness) and the time spent in movement phases during (i) STS and (ii) STW are correlated strongly to walking speed and/or step length ratio in people after stroke (aim two), Pearson's bivariate correlations were calculated for:

a) walk speed with movement phase duration and fluidity measures of STS;
b) walk speed with movement phase duration and fluidity measures of STW;
c) step length ratio with movement phase duration and fluidity measures of STS;
d) step length ratio with movement phase duration and fluidity measures of STW.

All tests were evaluated using a significance level of 0.05. Correlations were considered to be strong if 0.6 or above, moderate at a value of 0.4–0.6 and weak if 0.4 or below, suitably reversed for negative values (26).

## Results

### Participant Flow

**Figure 1:**
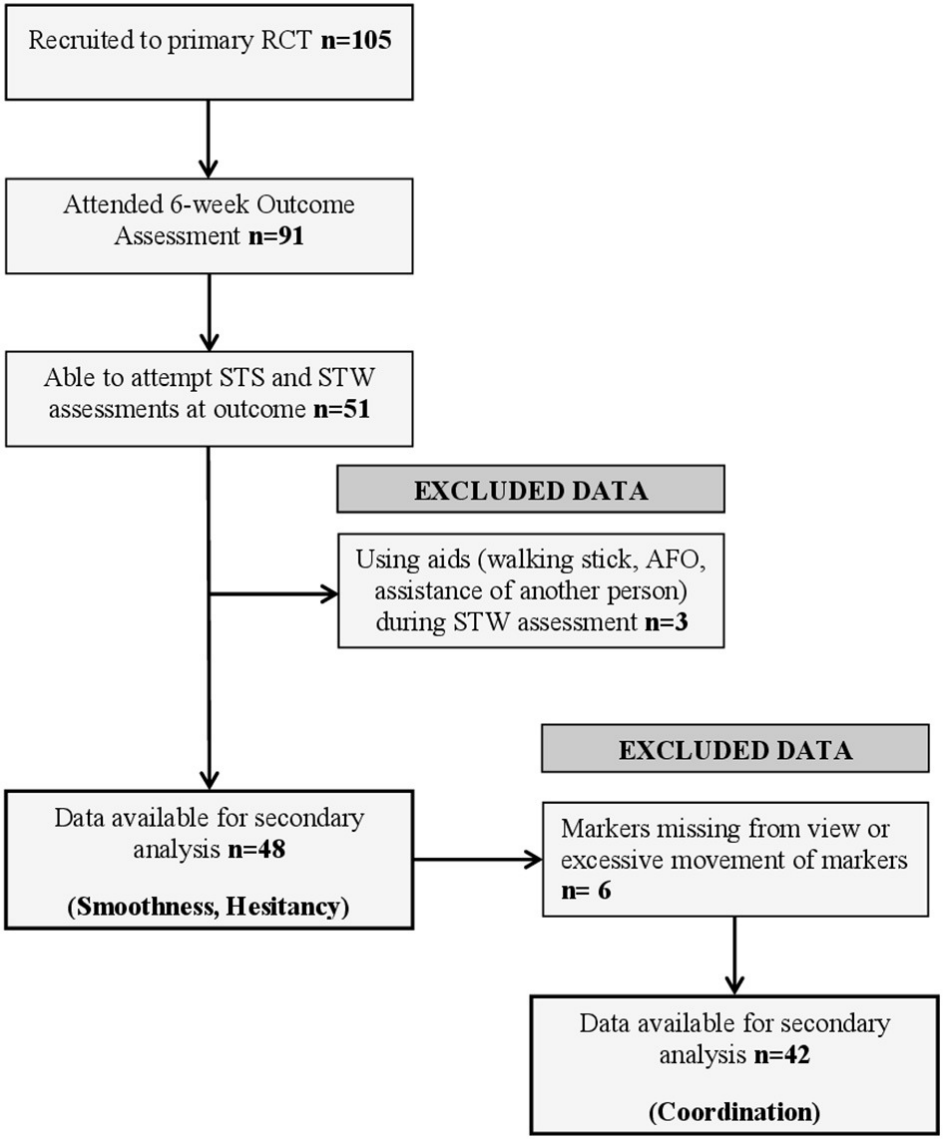
Flow chart of participant inclusion in this analysis.

Figure 1 illustrates participant flow through the analyses, with reasons for exclusion. A total of 105 participants were recruited into the original randomized controlled trial; of these, 91 attended the 6-week assessment from which data for this study were collected. At this assessment, 51 participants were able to attempt both STS and STW assessments. Three datasets were excluded because participants used walking aids or had physical assistance from another person. Consequently, 48 datasets were available for assessment of movement phase duration, smoothness and hesitation during STS and STW. A further six sets of data were excluded from coordination analysis because of large gaps in marker trajectories or excessive movement of cluster markers during the assessments. It was not possible to determine movement phases using our custom scripts for one participant during the STS task meaning 47 sets of data were available for analysis. Three participants completed STS and STW assessments but were unable to walk 3 m unaided, these participants were assigned a walking speed of 0 ms^−1^ and their step length ratio was treated as missing data.

The clinical characteristics of included participants are provided in Table 1. In summary, at outcome assessment participants' mean age was 65 years, their mean number of days post-stroke was 64 and they had a mean Functional Ambulatory Categories (FAC) score of 4.10/5. The average walking speed for all participants was 0.53 ms^−1^ ± Standard deviation (SD) 0.30 ms^−1^ with a step length ratio average of −0.03 ± SD 0.19.

**Table 1:** Clinical characteristics of participants included in this analysis.

	**Total sample (*n* = 48)**
**Participant demographics**	
Gender = male, *n* (%)	28 (57.1)
Age (years)^*^, mean ± SD	64.67 ± 15.58
**Clinical characteristics**	
Time since stroke (days)^*^, mean ± SD	63.56 ± 27.55
Type of stroke = infarct, *n* (%)	39 (81.25)
Paretic side = right, *n* (%)	30 (62.5)
**Baseline clinical scores**	
FAC (score/5) mean ± SD	4.10 ± 0.63
MRMI (score/40) mean ± SD	36.58 ± 3.94

* *Time at Outcome Assessment*.

### Comparison of Fluidity and Movement Phases Between STS and STW

Table 2 shows comparisons between STS and STW for both fluidity and movement phases. There was no significant difference in the mean overall time taken to complete the tasks of STS (*M* = 3.27 s ± SD 0.85 s) and STW (*M* = 3.23 s ± SD 2.00 s) [95%CI −0.05 (−0.43, 0.53), *p* = 0.84]. Analysis according to the pre-defined movement phases of STS and STW demonstrated that Phase 1 (*flexion momentum*, from initiation of movement until peak vertical velocity) lasts significantly longer during STS (*M* = 1.74 s ± SD 1.45 s) than in STW (*M* = 1.13 s ± SD 1.03 s) [95% CI −0.61 (−0.36, −0.86) *p* ≤ 0.0001].

**Table 2:** Comparison of fluidity and duration of movement phase variables between STS and STW [Mean (SD)].

**Fluidity measure**	**STS**			**STW**			***t*****-test**	
	***n***	**Mean**	**(SD)**	***n***	**Mean**	**(SD)**	**Difference (SD)**	***p*-value**
Smoothness (inflection count)	48	55.28	6.63	48	68.43	11.48	13.13 (9.08, 17.21)	**<0.001**
Hesitation (temporal overlap, %)	48	23.54	14.13	48	14.27	8.65	−9.27 (−14.29, −4.26)	**<0.01**
Coordination 1 (C1) paretic (temporal overlap, %)	20	7.38	5.49	34	15.39	12.99	−13.48 (−21.35, −5.60)	**<0.01**
Coordination 1 (C1) non-paretic (temporal overlap, %)	21	7.53	4.33	38	15.36	11.17	−8.76 (−13.11, −4.42)	**<0.01**
Coordination 2 (C2) paretic (temporal overlap, %)	NA	NA	NA	30	−14.11	15.93	NA	NA
Coordination 2 (C2) non-paretic (temporal overlap, %)	NA	NA	NA	10	−14.44	17.02	NA	NA
**Movement phases**								
Overall time (s)	47	3.27	0.85	48	3.23	2.00	−0.05 (−0.43, 0.53)	0.84
Phase 1 time (s)	47	1.74	1.45	48	1.13	1.03	−0.61 (−0.36, −0.86)	**<0.0001**
Phase 2 time (s)	47	−0.14	0.80	48	−0.14	0.86	0.03 (−0.39, 0.33)	0.87
Phase 3 time (s)	47	1.68	0.85	48	1.36	1.30	−0.36 (−0.03,0.75)	0.07
Phase 4 time (s)	NA	NA	NA	48	0.74	0.18	NA	NA

*The bold values refer to statistical significance*.

Fluidity measures show that STS had a statistically significant lower smoothness value [STS *M* = 55.28 inflections ± SD 6.63 inflections, STW *M* = 68.43 inflections ± SD 11.48 inflections, 95% CI 13.13 (9.08, 17.21) *p* ≤ 0.0001] indicating less inflections in the jerk signal and a smoother movement overall. Hesitation values show that STS is a more hesitant movement than STW with participants spending a significantly greater percentage of time in the transition between maximum forward velocity and the maximum upward acceleration [STS *M* = 23.54% ± SD 14.13%, STW *M* = 14.27% ± SD 8.65%, 95% CI-9.27 (−14.29, −4.26) *p* ≤ 0.01]. During STS, C1 in both paretic (*M* = 7.38% ± SD 5.49%, *p* ≤ 0.01) and non-paretic (*M* = 7.53% ± SD 4.33%, *p* ≤ 0.01) sides is shortened when compared to C1 in STW (paretic *M* = 15.39% ± SD 12.99%, non-paretic *M* = 15.36% ± SD 11.17%). This shows that the percentage of normalized time spent in between the events of the end of initial hip flexion, prior to seat off, and the start of knee extension is reduced for STS compared to STW indicating a more coordinated movement. Both C1 and Hesitation occur in movement Phase 1 of STS and STW.

### Relationship Between STS and STW With Walk Speed

The relationships between walking speed, fluidity measures and movement phase durations of STS and STW are provided in Table 3. Although statistical significance was reached for some variables none showed a strong correlation with walking speed (*r* = −0.51 to *r* = 0.42).

**Table 3:** Correlations between walking speed and measures of fluidity and duration of movement phases during STS and STW.

**Fluidity measure**	**STS**		**STW**	
	**Correlation**	***p*-value**	**Correlation**	***p*-value**
Smoothness (inflection count)	−0.34	**0.02**	0.42	**<0.01**
Hesitation (temporal overlap, %)	0.19	0.19	−0.08	0.58
Coordination 1 (C1) paretic (temporal overlap, %)	0.05	0.85	0.24	0.16
Coordination 1 (C1) non-paretic (temporal overlap, %)	0.23	0.32	0.36	**0.02**
Coordination 2 (C2) paretic (temporal overlap, %)	NA	NA	−0.35	0.06
Coordination 2 (C2) non-paretic (temporal overlap, %)	NA	NA	−0.51	0.13
**Movement phases**				
Overall time (s)	−0.41	**<0.001**	−0.31	**0.03**
Phase 1 time (s)	−0.42	**<0.001**	−0.15	0.31
Phase 2 time (s)	0.28	0.06	0.25	0.08
Phase 3 time (s)	−0.37	**0.01**	−0.51	**<0.001**
Phase 4 time (s)	NA	NA	−0.28	**0.05**

*The bold values refer to statistical significance*.

The correlations that were statistically significant indicate moderate to weak relationships between walking faster and shorter duration of both the STS and STW tasks, *r* = −0.41, *p* ≤ 0.01 and *r* = −0.31, *p* = 0.03, respectively. Faster walking also showed a moderate to weak correlation with: STS Phase 1 (*r* = −0.42, *p* ≤ 0.01), STS Phase 3 (*r* = −0.37, *p* = 0.01), STW Phase 3 (*r* = −0.51, *p* = 0.00) and STW Phase 4 (*r* = −0.28, *p* = 0.05).

A statistically significant, weak relationship was identified between greater smoothness and higher walking speed for STS (*r* = −0.34, *p* = 0.02). The opposite relationship was found for STW with a significant but moderate correlation (*r* = 0.42, *p* ≤ 0.01) between less smooth movement and higher walking speed.

No other fluidity measures for STS were correlated significantly to walking speed. For STW a weak relationship was found between C1 of the less-paretic lower limb and greater walking speed (0.36, *p* = 0.02).

### Relationship Between STS and STW With Step Length Ratio

Table 4 demonstrates the relationship between step length ratio; duration of movement phases and fluidity measures from STS and STW. All correlation coefficients were weak (*r* = −0.27 to *r* = −0.21) and none were statistically significant.

**Table 4:** Correlations between step length ratio during walking and measures of fluidity and duration of movement phases during STS and STW.

**Fluidity measure**	**STS**		**STW**	
	**Correlation**	***p*-value**	**Correlation**	***p*-value**
Smoothness (inflection count)	−0.01	0.97	−0.04	0.79
Hesitation (temporal overlap, %)	−0.11	0.49	−0.25	0.10
Coordination 1 (C1) paretic (temporal overlap, %)	0.03	0.89	−0.09	0.62
Coordination 1 (C1) non-paretic (temporal overlap, %)	0.05	0.82	−0.01	0.95
Coordination 2 (C2) paretic (temporal overlap, %)	NA	NA	0.06	0.77
Coordination 2 (C2) non-paretic (temporal overlap, %)	NA	NA	−0.25	0.49
**Movement phases**				
Overall time (s)	−0.06	0.72	−0.14	0.35
Phase 1 time (s)	0.02	0.88	−0.18	0.25
Phase 2 time (s)	−0.17	0.26	0.21	0.17
Phase 3 time (s)	0.02	0.88	−0.27	0.07
Phase 4 time (s)	NA	NA	−0.24	0.11

## Discussion

### Summary of Findings

Our results do not support the hypothesis that measures of movement fluidity and movement timing during STS and STW are correlated strongly with walking speed and step length symmetry in people early after stroke.

The study found that whilst people who were a mean of 64 days after stroke took the same amount of time to complete both STS and STW, participants took significantly longer to complete the flexion momentum phase of STS than of STW (aim 1). Differences between performance of the two tasks were also found for movement fluidity. Specifically, compared to STW, the STS task was performed significantly smoother but with greater hesitancy and greater hip/knee coordination (aim 1). No strong relationship was found for stroke survivors between: walking speed and STS or STW; walking speed and duration of STS or STW or their constituent phases; step length ratio during walking and STS or STW; or, step length ratio during walking and STS or STW (aim 2). However, significant weak to moderate relationships indicated that stroke survivors who walked faster may also: perform the STS task more smoothly, but perform STW less smoothly and have reduced hip/knee coordination on their non-paretic leg during STW. Unsurprisingly, faster walkers also take less time to complete STS and STW; they spend less time in the flexion momentum phase of STS and have shorter durations of Phase 3 (*extension momentum*) of STS and STW and Phase 4 (*stance*) of STW.

In summary, our findings indicate that the lack of a strong relationship between walking speed/step length symmetry to movement fluidity and duration of STS and STW means that all three tasks require distinct training after stroke. No one task is superfluous for stroke rehabilitation.

### The Differences Between Movement Fluidity and Duration of Phases Between STS and STW

Significantly greater hesitation was observed during STS than during STW in this group of people early after stroke. This finding is similar to previous findings that hesitation is greater during STS than STW in healthy younger adults (25) despite the variation in the description and calculation of hesitation between studies. As the events of hesitation (maximum forward velocity and maximum upward acceleration) both occur around the end of Phase 1 of movement, the *flexion momentum*, these data indicate that hesitation is likely contributing factor to the longer Phase 1 of movement seen in STS compared to STW. A prolonged Phase 1 has previously been described in studies examining STW in stroke survivors when compared to healthy adults; here stroke survivors spent a greater amount in Phase 1 because of increased time spent in hip flexion (7). A lengthened Phase 1 of movement is also seen in older adults, when compared to younger adults attributed to an increased angle of trunk flexion (27). Hesitation may be a critical time window in which balance is tightly regulated to create the breaking impulse previously identified as an important differentiation between these tasks in healthy adults (25, 28).

STS was found to be both a smoother and a more coordinated movement than STW. This likely reflects the less challenging nature of the STS task without asymmetric unloading of the swing leg, gait initiation and initial steps and the balance perpetuations associated with these actions. The biomechanical events measured to determine C1 appear to occur around the transition between movement Phases 1 and 2 indicating that in stroke survivors, preparation for seat-off in STW takes longer than in STS. This may reflect the time required for the medio-lateral ground reaction force and unloading of the swing leg seen in STW but not in STS in healthy adults (25, 28). It is interesting that when compared to previous data from healthy adults, who begin knee extension before hip flexion ends (24), stroke survivors here show an inverse pattern of movement during C1, demonstrating an inability to begin knee extension until after the end of hip flexion.

This assessment of STS and STW in the same group of stroke survivors shows that the functional tasks of STS and STW create distinct biomechanical characteristics which can be identified using sensitive, objective measures of fluidity and timing within movement phases. The identification of these characteristics may be indicative of the different movement intentions and therefore the motor planning strategies required for the seemingly similar tasks of STS and STW. This clearly demonstrates that it is not possible to assess recovery post-stroke with just one task even if that task shows clear similarities to another. Similarly, interpretations of STS data cannot be made in relation to a STW task and vice-versa.

### The Relationship of Fluidity Measures to Walk Speed

Previous publications have described associations between total STW duration and walking speed (*r* = −0.42, *p* < 0.01) in older adults (29) and a fluidity index with a 10 m timed walk (*r* = −0.73, *p* < 0.0001) in chronic stage stroke survivors (30). The data in our study show much weaker correlations between walking speed and STS smoothness (*r* = −0.34, *p* = 0.02), STW smoothness (*r* = 0.42, *p* < 0.01), STW C1 of the non-paretic leg (*r* = 0.36, *p* = 0.02), overall time to complete STS (*r* = −0.41, *p* ≤ 0.01), overall time to complete STW (*r* = −0.31, *p* = 0.03), time to complete Phases 1 (*r* = −0.42, *p* < 0.01) and 3 (*r* = −0.37, *p* = 0.01) of STS and time to complete Phases 3 (*r* = −0.51, *p* < 0.01) and 4 (*r* = −0.28, *p* = 0.05) of STW. However, whilst it is important to acknowledge findings from similar work in the field, direct comparisons with these existing studies are challenged by use of an older adult study population without specific neurological impairment (29) and use of the previously discussed Fluidity Index that perhaps does not reflect the complexity required to measure motor control strategies in people early after stroke, as we have done here (30).

In this analysis, the overall speed at which the functional movements of STS and STW are completed shows moderate correlation to the speed at which a stroke survivor can walk. These measures are a simple measure of functional ability but cannot be interpreted in relation to neuromuscular restitution. The duration of movement Phases 1 and 3 in STS and 3 and 4 in STW also show a moderate relationship to walking speed. The duration of Phases 3 and 4 during STW have been previously identified as prolonged in stroke survivors when compared to healthy control participants (30). The correlation of STW Phases 3 and 4 may suggest that both gait initiation and initial step of STW may reflect aspects of walking. However, the nature of gait initiation from a seated position in STW is likely a more challenging and dynamic movement than walking at a self-selected speed, in a straight line, across a level surface. Although significance was not reached it is interesting to note that for both STS and STW the duration of Phase 2, i.e., *seat-off* , shows the opposite pattern to the rest of the movement phases. Here a slower movement is seen, which may be indicative of the importance of motor control around the crucial event of seat-off where optimum balance is essential.

Measures of movement fluidity during STS and STW showed a moderate relationship between the ability to STS in a smooth movement and walking speed whereas the opposite was found for STW. This may be due to the decision made here to collect STS data until the peak vertical displacement of the clavicle marker whereas the STW data is collected until foot contact of the second step. As a result, the STW data encompasses gait initiation and the initial two steps which require rapid acceleration and deceleration of the COM not required for a STS movement. A smoother STW may be seen in those participants who essentially STS, pause and then tentatively start to walk whilst maintaining tight control due to lack of confidence or balance. Significant breaking impulses prior to seat-off have been previously identified in stroke survivors performing a STW task (31) which may contribute to less smooth movement of STW compared to STS, further investigation is required to confirm this.

The only other fluidity measure to show a relationship to walking speed is that of C1 (the temporal overlap between the knee and hip during rising). Here a larger value, indicating less coordination, shows a moderate relationship to walking speed. C1 has previously been investigated during STS (6) and the stepping leg of STW (24). Here we made the decision that, where marker visibility allowed, we would investigate C1 of both the stepping and stance legs. In this analysis, almost all participants used their paretic leg to take the initial step and therefore, with few exceptions, all of the C1 data from STW relates to the stance leg which has not previously been investigated. The greater value seen in C1 during STW may indicate a different motor strategy to that used in STS, perhaps the preparation for/beginning of forward propulsion through the stance leg.

The absence of any identified strong relationships between the measures of walking speed, fluidity measures and timing within movement phases during either STS or STW demonstrates the complexity of assessing recovery after stroke. Although relationships between the functional tasks of STS, STW and walking had previously been suggested, the data in this study indicates that any relationship is, at best, tenuous. Walking speed is simple and easy to measure; however, its usefulness in the assessment of motor recovery in stroke survivors is limited. Speed can be achieved through a variety of compensatory techniques and it is probably a better indicator of balance and confidence than recovery. Speed of STS, STW or their movement phases showed the strongest relationship to walking speed of all the measures used in this study. This may indicate that these commonly used measures of STS and STW are, like walking speed, just a measure of functional ability without the sophistication to measure the underlying reasons for a faster movement.

Fluidity measures of smoothness, hesitation and coordination were developed with the aim of measuring the ability to move in a controlled and fluid way without rapid changes. Both hesitation and coordination measure normalized time between biomechanical events; however, unlike movement phases, the events used were chosen with the specific aim of providing an objective measure of a therapists subjective observation- that improving fluidity could improve function (32). This is a clear demonstration of the need to carefully consider the mechanisms behind assessment tasks to fully appreciate what is being measured.

### The Relationship of Fluidity Measures to Step-Length Ratio

No relationship was found for any of the measures described when compared to step length symmetry. A fluid STS or STW is thought to be indicative of motor control (9); however, there is a lack of evidence for measures that can identify motor control during gait. Step length symmetry was chosen as a comparator in this study because of the potential to provide information regarding movement quality which cannot be discerned from walking speed. The lack of relationship between gait symmetry and walking speed (33, 34) further strengthens the idea that spatiotemporal symmetry measures different aspects of walking from those measured by velocity.

### Implications of Findings to the Measurement of Neuromuscular Recovery After Stroke

Walking, STS and STW clearly have points of commonality. Both STS and STW involve forward lean of the trunk and bilateral lower limb extension to rise from a seated position to bipedal standing. Likewise, STW and walking involve transition of bodyweight between the supporting feet whilst moving body position in space. Consequently, there is an expectation of relationships between some elements of the three movement tasks and therefore some transferability of rehabilitation training benefit between the tasks. However, the results of this study indicate that, in a group of early stroke survivors there are: significant differences between STS and STW for movement fluidity (smoothness, hesitation and coordination); only moderate relationships at best between walking speed and: movement fluidity during either STS or STW; duration of STS or STW and its phases and no relationship between symmetry (step length ratio) and the tasks of STS and STW. The different movement characteristics of the three tasks likely mean that measures of any one of these three tasks cannot be used to infer ability to perform either of the others. Likewise, it follows that rehabilitation needs to consider separate training of the three tasks after stroke.

Specific training of the separate tasks of STS, STW, and walking is also indicated by knowledge of the muscle synergies (activation patterns of muscles used) that produce the movement required to undertake complex movement tasks (35–37). Muscle synergies have been described as the building blocks of complex movements and vary depending on the movement task in people who do not have a stroke lesion (35–37). Pertinent to the current study is that STS and walking involve the use of different muscle synergies (38, 39) and presumably STW contains elements of both. Consequently, rehabilitation to restore pre-stroke body function, that identified in people without a stroke lesion (2), should focus on the specific movement tasks required for independent living. Furthermore, measures to assess whether the pre-stroke body function is being restored should also be specific to the task being trained. The work presented here has expanded knowledge on the content and use of such measures- our measures of fluidity were directly informed by and expanded on previous valuable work on a Fluidity Index by Dion et al. (9). Where this previous Fluidity Index was based on CoM velocity in one direction, we have represented the complexity of the task in an attempt to identify areas that might be targeted by therapists (25) and applied our measures in this current work to evaluate important functional tasks in a large group of people in the early weeks after stroke.

Two messages are clear from this analysis: firstly, that assessment of movement after stroke is about more than just walking speed or even walking task performance. A range of functional tasks are required to gain a full understanding of recovery and no one task should predominate over another. Measuring seemingly similar tasks such as STS and STW is not superfluous as the differing nature and ultimate intention of the tasks makes each challenging in different ways. Secondly, mechanisms behind the assessment measures must be thoroughly considered and it is this that should determine the appropriate task and assessment.

### Methodological Considerations

Our study had several limitations which should be considered in the interpretation of the methods and results. The main limitation is that, whilst the intention was to make comparisons of the different functional tasks of STS, STW, and walking there are not truly comparable measures available for the tasks. Every effort was made to ensure measures between STS and STW were as similar as possible, but the different natures of the tasks made complete transferability impossible. This particularly affected the comparison of smoothness between the tasks due to the different end point of each task. We also recognize that allowing participants to use one or both hands as they rose, if this was required for safety reasons led to some potentially slightly altered movement strategies, though this did enable pragmatic representation of the strategies adopted here in this clinically representative population. The other limitation to this study is the amount of lost data from the original SWIFT Cast Trial. These measures proved difficult to capture in a clinical population early after stroke, some participants were unable to carry out the tasks, some carried out the task but used walking aids or received assistance, which made their inclusion in this analysis impossible due to a lack of standardization. Marker visibility was restricted by stroke related postures and movement along with the need to maintain a researcher close to the participant for safety. As a result, we were unable to consistently collect COM data and had to instead use a single clavicle marker to reflect the movement of the trunk. Finally, some data was lost due to unusual movement patterns which could not be identified by the custom-made script. Many versions were written to try to account for every eventuality but the variation in movement exhibited by stroke survivors could not be completely expected and therefore it was not always possible to identify events using a script.

The methodological strengths of the study are that it used kinetic and kinematic data to explore established measures during the functional tasks of STS, STW and walking. Importantly, these data were collected from the same group of stroke survivors, at the same assessment, which enabled investigation of how the ability to perform one functional task may or may not influence another. To the best of our knowledge this is the first study to examine this. Although it was not possible to include all the data collected in this study a sample size of 48 is relatively high in comparison to many other biomechanical studies. This, coupled with the fact that participants were on average just 64 days post-stroke and recruited from a clinical population, means that these data can make a substantial contribution to knowledge about measures of assessment and rehabilitation techniques early after stroke.

## Conclusion

The main findings of this study are that: (i) different movement intentions between STS and STW create distinct biomechanical characteristics which can be identified using sensitive objective measures of fluidity and movement phases but (ii) despite findings of statistical significance there are no strong relationships between the functional tasks of STS and STW with walking speed (iii) symmetry during walking, measured by step-length symmetry, shows no relationship to any measures of fluidity or movement phases during STS and STW.

## Data Availability Statement

The raw data supporting the conclusions of this article will be made available by the authors, without undue reservation.

## Ethics Statement

The studies involving human participants were reviewed and approved by National Research and Ethics Service (Reference 09/H0310/87) registered on the ISRCTN database https://doi.org/10.1186/ISRCTN39201286. The patients/participants provided their written informed consent to participate in this study.

## Author Contributions

EC: conceptualization of this study, methodology, data collection, data processing, writing-original draft preparation, writing-reviewing, editing, and approval of submitted version. TS: methodology, script writing for data analysis, writing-reviewing, editing, and approval of submitted version. VP: conceptualization of this study, data collection, writing-original draft preparation, writing-reviewing, editing, supervision, and approval of submitted version. AC: data analysis, writing-reviewing, editing, and approval of submitted version. AK and UU: data collection, data processing, writing-reviewing, editing, and approval of submitted version. PR: writing-reviewing, editing, and approval of submitted version. JS: data collection, data processing, writing-reviewing, and approval of submitted version. NH: conceptualization of this study, methodology, data processing, writing-original draft preparation, writing-reviewing, editing, supervision, and approval of submitted version. All authors contributed to the article and approved the submitted version.

## Conflict of Interest

The authors declare that the research was conducted in the absence of any commercial or financial relationships that could be construed as a potential conflict of interest.
